# Cost-effectiveness of a potential vaccine for Coccidioides immitis.

**DOI:** 10.3201/eid0705.010505

**Published:** 2001

**Authors:** A E Barnato, G D Sanders, D K Owens

**Affiliations:** University of Pittsburg, Division of General Internal Medicine, Pennsylvania 15213, USA. barnato@post.harvard.edu

## Abstract

Coccidioidomycosis, a systemic fungal infection, affects Americans living in the Southwest. We evaluated the cost- effectiveness of a potential vaccine against Coccidioides immitis. Using a decision model we developed, we estimate that among children, vaccination would saved 1.9 quality-adjusted life days (QALD) and $33 per person. Among adults, screening followed by vaccination would save 0.5 QALD per person and cost $62,000 per quality adjusted life year gained over no vaccination. If the birth cohort in highly endemic counties of California and Arizona were immunized in 2001, 11 deaths would be averted and $3 million would be saved (in net present value) over the lifetime of these infants. Vaccination of adults to prevent disseminated coccidioidomycosis would provide a modest health benefit similar in magnitude to other vaccines but would increase net expenditures. Vaccination of children in highly endemic regions would provide a larger health benefit and would reduce total health care expenditures.


### Cost-Effectiveness of a Potential Vaccine for *Coccidioides immitis*

**Amber E. Barnato,* Gillian D. Sanders,† and Douglas K. Owens†‡**
*University of Pittsburgh, Pittsburgh, Pennsylvania, USA; †Stanford University, Stanford, California, USA; and ‡VA Palo Alto Health Care System, Palo Alto, California, USA

Coccidioidomycosis, a systemic fungal infection, affects Americans living in the Southwest. We evaluated the cost-effectiveness of a potential vaccine against *Coccidioides immitis*. Using a decision model we developed, we estimate that among children, vaccination would save 1.9 quality-adjusted life days (QALD) and $33 per person. Among adults, screening followed by vaccination would save 0.5 QALD per person and cost $62,000 per quality adjusted life year gained over no vaccination. If the birth cohort in highly endemic counties of California and Arizona were immunized in 2001, 11 deaths would be averted and $3 million would be saved (in net present value) over the lifetime of these infants. Vaccination of adults to prevent disseminated coccidioidomycosis would provide a modest health benefit similar in magnitude to other vaccines but would increase net expenditures. Vaccination of children in highly endemic regions would provide a larger health benefit and would reduce total health care expenditures.

*Coccidioides immitis*, an infectious fungus, grows in the arid soil of the Central Valley of California, southern Arizona, and parts of Nevada, New Mexico, Utah, and Texas, as well as northern Mexico and parts of Central and South America. Regions endemic for *C. immitis* are home to approximately 20% of the U.S. population; an estimated 5 million persons live in the areas of highest endemicity (Figure 1) (1-3). Humans are infected by inhaling dust containing *C. immitis* arthroconidia. Dust storms (4,5) and activities associated with heavy dust exposure such as agricultural labor (6), excavating archeologic ruins (7), and military combat training (8,9) increase infection rates, total infectious load, and the proportion of symptomatic cases. Infection rates, as reflected by positive skin tests, have decreased since the 1940s and 1950s from as high as 8% per month to approximately 2% to 3% per year in highly endemic regions. This decrease is likely the result of reduced dust exposure attributable to lifestyle changes and urbanization. However, population growth in endemic regions has been steady and is projected to increase.

Recent epidemics in California and Arizona highlight the continuing public health threat and costs of coccidioidomycosis (10,11) and have led to efforts to develop a vaccine. An economic analysis of the 7,130 cases from 1991 to 1993 in California's Kern County demonstrated a cost to that county of $56 million (12). Because the U.S. training post for desert warfare is located in the Mojave Desert, an area endemic for *C. immitis*, a vaccine is a military as well as a civilian priority. A killed whole spherule vaccine that showed promise in animal trials was not well tolerated in humans (13). Current efforts of the Valley Fever Vaccine Project, a consortium of researchers funded primarily by the California Healthcare Foundation and the California Department of Health Services, focus on a number of antigens successful in mice models that may form the basis of a subunit vaccine (G. Rutherford, pers. comm.).

Despite the potential clinical value of a vaccine, the only study of the cost-effectiveness of a vaccination program was an Institute of Medicine (IOM) analysis for the purposes of setting priorities in vaccine development (14). The IOM concluded that vaccine development would cost more than $100,000 per quality-adjusted life year (QALY) gained, but included vaccine development costs and used a very simplified model. If the vaccine would not be cost-effective, current development efforts might be in vain. We present the results

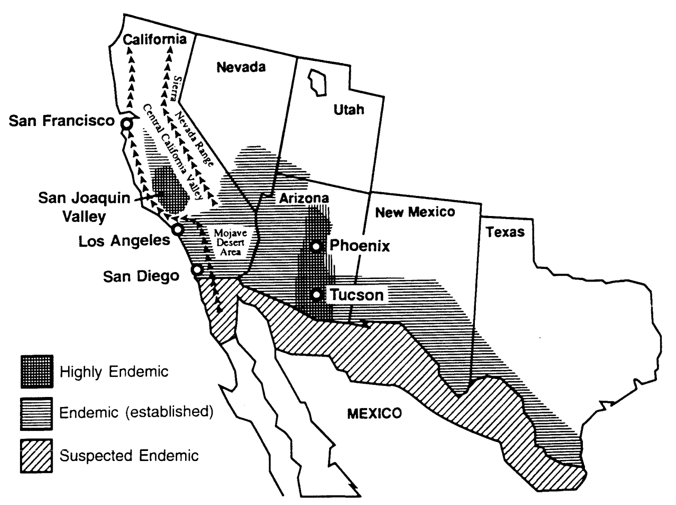

Figure 1. Areas in the United States endemic for *Coccidioides immitis*. Cross-hatching indicates the heavily disease-endemic area; single hatching indicates the moderately disease-endemic area. Reprinted with the author's permission from Kirkland (1).

Address for correspondence: Amber E. Barnato, University of Pittsburgh School of Medicine, Division of General Internal Medicine, 933W-MUH, 200 Lothrop Street, Pittsburgh, PA 15213, USA; fax: 412-692-4838; e-mail: barnato@post.harvard.edu of a detailed cost-effectiveness analysis of a potential vaccine against *C. immitis*.

### Data and Methods

We used a decision model to evaluate the health and economic consequences of withholding the vaccine, screening and vaccinating only those susceptible to infection (screening/vaccination), or vaccinating all eligible persons. Taking a societal perspective, we calculated the incremental cost-effectiveness of these strategies (formula in Appendix I, online; URL: http://www.cdc.gov/ncid/eid/vol7no5/barnato_appendix1.htm), and we discounted both costs and benefits at an annual rate of 3%. We performed one-way sensitivity analyses on all the model variables, as well as a three-way sensitivity analysis of the most sensitive variables, and best- and worst-case scenarios.

We based the estimates for input variables on the literature whenever possible (Appendix II Table). Our base-case estimates represent our judgment about the best estimate from the literature and discussion with experts. The ranges for costs represent variation by 25% above and below the base-case estimate, except where otherwise specified. The ranges for sensitivity analyses on quality-of-life estimates represent the 25th and 75th percentiles of patients' assessments of quality of life (15), except where otherwise specified. The authors had complete scientific and editorial independence from the funding agencies.

### Decision Model

We developed a Markov model (Figure 2) with Decision Maker software (version beta 0.99.11.14.0a, 2000, S. Pauker, et al., Boston, MA) (16,17) to track hypothetical cohorts of patients who either did not receive the *C. immitis* vaccine, received it, or received it only if their skin test was negative. Patients who received the vaccine were at risk for pain at the injection site, mild to moderate fever, and anaphylaxis without death. Vaccinated patients were at decreased risk for extrapulmonary dissemination after primary infection. Patients neither immune from previous infection nor successfully vaccinated were at risk for *C. immitis* infection and serious sequelae. All patients were at risk for dying from other causes (18). We followed cohorts until death, with a Markov cycle length of 1 month.

### Patient Population

We used epidemiologic and demographic data from two California counties (Kern and Tulare) and eight Arizona counties (Cochise, Gila, Graham, Maricopa, Pima, Pinal, Santa Cruz, and Yavapai) as proxies for the population features of highly endemic regions (2,19; Internal Revenue Service, unpub. data). We used two cohorts representing the weighted average age and prior probability of naturally acquired immunity among children (ages $\leq$17) and adults (ages 18 to 65) in 10 highly disease-endemic counties. New residents with no natural immunity were added to these cohorts. Children had an average age of 8.85 years, and 14.5% were naturally immune; adults had an average age of 39.51 years, and 47.5% were naturally immune.

### Clinical Manifestations

Smith's classic studies in military recruits stationed in disease-endemic regions during World War II established that 60% of infected persons have no symptoms and 40% have a flulike illness (20). Asymptomatic infection and symptomatic infection with recovery confer probable lifelong immunity with a positive delayed-type hypersensitivity skin-test reaction to coccidioidin or spherulin. However, a fraction of per-

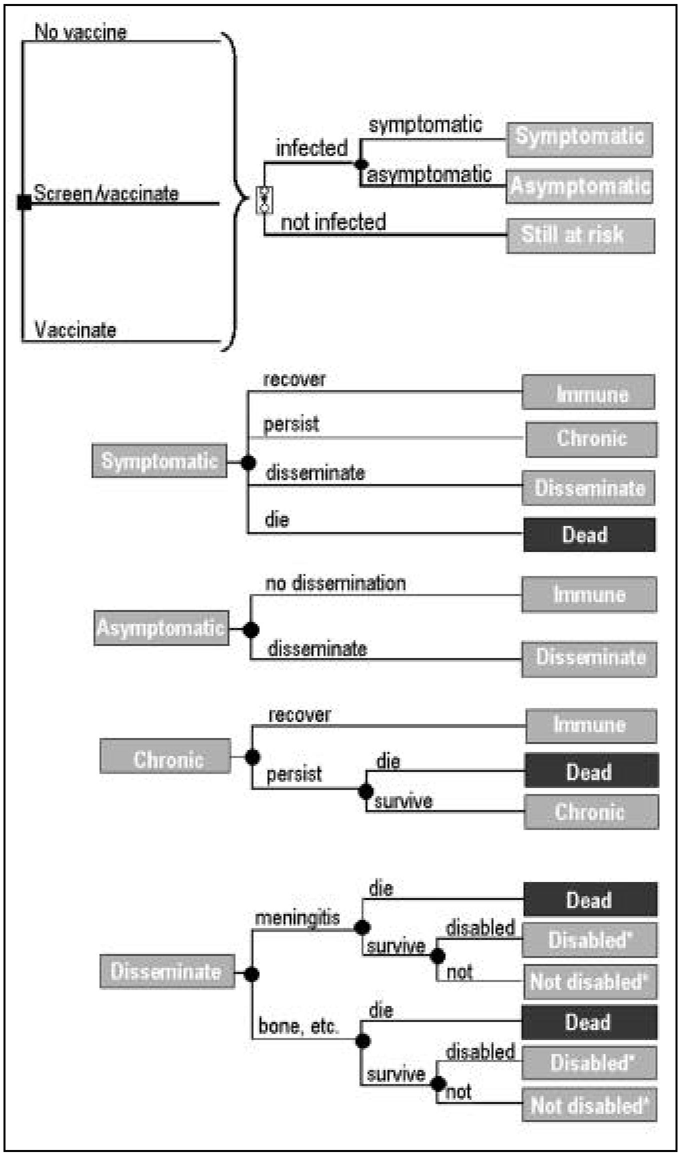

Figure 2. Schematic representation of the decision model and decision model subtrees. The square node represents a decision to use one of the three strategies: no vaccination, vaccination of susceptible persons identified through a screening skin test, or vaccination of all persons. Circles represent chance nodes. After a strategy is chosen, the patient enters a Markov tree (denoted by a rectangle containing circles connected by an arrow). The Markov tree represents clinical events that can occur during each 1-month period as a patient is followed until death. Subtrees show events that may occur to patients during a 1-month cycle. Dissemination subtree: The site of extrapulmonary dissemination can be to the brain (meningitis) or elsewhere (e.g., bone). The outcome of dissemination can be death from other causes, death from disseminated coccidioidomycosis, or survival. If the patient survives, he or she can survive in a disabled or nondisabled state. Each month thereafter, the patient is at risk for relapse. Chronic subtree: Each month a patient with chronic pulmonary coccidioidomycosis can die from other causes, remain infected, die from chronic pulmonary infection, or be cured and rendered immune. Immune subtree: On any given month, a patient who is immune to infection can die of other causes or remain alive and immune to coccidioidomycosis.
*At risk for dissemination relapse.

sons do not have simple self-limited disease and instead more serious illness develops, such as respiratory failure, chronic pneumonia, and extrapulmonary dissemination (20-23). We present more clinical detail in Appendix I, online, only (at URL:www.cdc.gov/eid/v7n5/barnato-appendix1.htm) and summarize probability estimates, costs, and utilities in the Appendix II Table.

### Vaccine and Skin-Test Characteristics

Animal trials of the vaccine rely on intraperitoneal or intranasal challenge with large loads of *C. immitis*. The outcome measure is survival or death from disseminated disease. Because the mechanism of action of the putative vaccine is not known, we conservatively assumed that vaccination prevents extrapulmonary dissemination but not primary infection. We evaluated this assumption in a sensitivity analysis.

We assumed that all vaccinated persons comply with 3 doses in 1 year and all develop an antibody response. We assumed that the vaccine reduced the probability of dissemination by 75%, with lifelong duration, based on experience with hepatitis B vaccine, a widely used subunit vaccine. We examined the effects of compliance with each strategy and the effect of waning immunity.

Using data from hepatitis B vaccine as a proxy, we assumed that 25% of patients would experience mild side effects such as local arm pain, fever, and nausea and that 1 in 600,000 patients would experience nonfatal anaphylaxis requiring hospitalization (24).

For screening before vaccination, studies suggested a sensitivity of 70% and a specificity of 90% for the spherulin intradermal test (25-30).

### Quality of Life

Our model included intermediate health states that may be associated with decrements in quality of life. To adjust for such decrements, we included quality adjustments in our model (Appendix II Table). Because there are no quality-of-life studies for coccidioidomycosis, we used proxy health state utilities in published studies or clinical judgment. All illness utilities were multiplied by age-specific “healthy” utilities to account for the age at which an illness was contracted. We assumed that primary pulmonary infection causes a substantial heath status decrement only in those who are sick enough to be diagnosed. Among those with primary pulmonary disease that goes undiagnosed, we assigned a health state utility equal to well for this illness episode, but accounted for lost time from work due to illness (see Costs, below). For the short-term vaccine side effects, we capture quality-of-life impact as a decrease in utility of one-tenth of a quality-adjusted day. The base case and ranges for these quality-of-life adjustments reflect our judgment about the impact of these episodes on patients. We evaluated the effects of these utility assumptions in sensitivity analyses.

### Costs

### Direct Medical Costs

All costs are presented in 2000 U.S. dollars (Appendix II Table). For costs of inpatient care, we used cost-adjusted charges from the 1996 Nationwide Inpatient Sample of the Healthcare Cost and Utilization Project (31). For outpatient services, we used the 2000 Medicare national physician fee schedule (32). For outpatient services not listed in the Medicare fee schedule, we used reimbursement received by Kern County service providers (R. Talbot, pers. comm.) We assumed that patients severely disabled by coccidioidal meningitis would require home support and used the average payment per Medicare home health beneficiary as a proxy for this cost (33).

### Vaccine and Other Costs

In our base-case analysis, we assumed the cost of the vaccine was $180. We chose this value for the base case because it is the reimbursed fee for the three-shot hepatitis B series, an existing subunit vaccine. We examined a broad range of vaccine costs ($100 to $400) in sensitivity analyses.

For circumstances in which quality-of-life changes do not capture the inconvenience of illness or medical care, we included time costs, as noted in the online Appendix I.

### Results

### Prevention of Illness and Death

We calculated the number of cases of disseminated disease and deaths per 100,000 persons that would be prevented over the lifetime of each cohort if children and adults were immunized, assuming rates of vaccine coverage of 40%, 60%, 80%, and 100% (Table 1). For example, if 60% of children were vaccinated, 93 cases of dissemination, 64 cases of disability, and 7 deaths would be averted and $2 million saved per 100,000 population. If 60% of adults underwent screening followed by vaccination, 38 cases of dissemination, 24 cases of disability, and 2 deaths would be prevented at a cost of $5.4 million per 100,000 population.

Table 1. Lifetime cases of dissemination, disability, and death prevented and costs per 100,000 population if children are vaccinated and adults are screened, then vaccinated

| | Dissemination prevented | | Disability prevented | | Deaths prevented | | Net cost ($ millions) | |
|---|---|---|---|---|---|---|---|---|
| Compliance | Children | Adults | Children | Adults | Children | Adults | Children | Adults |
| 100% | 154 | 63 | 106 | 39 | 12 | 4 | -3.3 | 9 |
| 80% | 124 | 50 | 85 | 32 | 10 | 3 | -2.6 | 7.2 |
| 60% | 93 | 38 | 64 | 24 | 7 | 2 | -2 | 5.4 |
| 40% | 62 | 25 | 42 | 16 | 5 | 2 | -1.3 | 3.6 |

### Costs and Effectiveness

We calculated the effectiveness, costs, and cost-effectiveness of each strategy in each population, expressed in Table 2 as the per-person cost and health benefit. Among children, vaccination saved 1.9 quality-adjusted life days (QALD) and $33 per person. Among adults, screening followed by vaccination saved 0.5 QALD per person and cost $62,000 per QALY gained over no vaccination. For adults, the incremental gain from vaccinating all persons compared with screening followed by vaccination contributed an additional 0.05 QALD at a cost of $235,000 per QALY gained.

### Sensitivity Analyses

We performed one-way sensitivity analyses over the ranges of all input variables listed in the table in Appendix II and on critical assumptions, such as the vaccine mechanism of action and target population. Among children, vaccination was no longer cost-saving at the lowest ranges of vaccine efficacy, infection rate, dissemination rate, long-term care cost for severe disability, and medical follow-up cost after nonmeningeal dissemination and chronic pulmonary disease; the vaccine also was not cost-saving in children when we used the highest ranges of vaccine cost, meningitis mortality, emigration, and discount rate among children. The counter-intuitive effect of meningitis deaths is due to the decrease in survivors subject to long-term care costs from post-meningitis disability. Among adults, sensitivity analyses that changed the cost-effectiveness ratio by ≥$40,000 per QALY gained included infection rate, vaccine effectiveness, discount rate, vaccine cost, dissemination rate, emigration rate, and office visit time. Only the vaccine cost changed the preferred strategy among adults. Below $106 per 3 doses, vaccination was preferred over screening/vaccination, with a cost-effectiveness ratio of $30,000 per QALY gained.

### Vaccine Duration

Our base-case analysis assumed lifetime immunity after vaccination, but vaccine protection may wane. If vaccine protection waned to zero in 15 years, vaccination saved 0.82 QALD per child and cost $47,300 per QALY gained, and screening/vacccination saved 0.28 QALD per adult and cost $165,500 per QALY gained.

### Vaccine Mechanism of Action

If the vaccine prevented primary infection rather than dissemination alone, vaccination saved 2.26 QALD and $46 per vaccinated child over no vaccination. Screening/vaccination saved 0.66 QALD per adult, costing $46,500 per QALY saved.

### Three-Way Sensitivity Analysis of Infection Rate, Vaccine Effectiveness, and Cost

Our base case assumed an infection rate of 2% per year, vaccine effectiveness of 75%, and a vaccine cost of $180. However, the vaccine may have some use in areas of lower endemicity. Also, because the vaccine does not yet exist, its cost and effectiveness are unknown. We present the effects of varying costs and effectiveness of the vaccine under two conditions: 0.5% infection rate per year and 2% per year (our base case of highly endemic regions) (Figure 3). In our base case for children, the $180 vaccine remains cost-saving down to an effectiveness of 65% and costs <$50,000 per QALY until vaccine effectiveness drops below 30%, confirming that the vaccine need not be highly effective to be cost-effective in

Table 2. Health and economic outcomes of vaccination strategies[a]

| Age | Strategy[b] | Life expectancy (years; days) | Incremental life expectancy[c] (days) | Quality-adjusted life expectancy (years; days) | Incremental quality-adjusted life expectancy[c] (days) | Cost ($) | Incremental cost[d] ($) | CE ratio ($/QALY)[e] |
|---|---|---|---|---|---|---|---|---|
| ≤17 | No vaccine | 28; 160.15 | - | 25; 172.98 | - | 35,196 | - | - |
| | Screen/ vaccinate | 28; 160.58 | 0.43 | 25; 174.66 | 1.68 | 35,187 | -9 | - |
| | Vaccinate | 28; 160.62 | 0.04 | 25; 174.84 | 0.18 | 35,163 | -24 | Dominates |
| 18-65 | No vaccine | 21; 272.68 | - | 18; 60.12 | - | 47,477 | - | - |
| | Screen/ vaccinate | 21; 272.82 | 0.14 | 18; 60.65 | 0.53 | 47,568 | 90 | 62,000 |
| | Vaccinate | 21; 272.84 | 0.02 | 18; 60.70 | 0.05 | 47,601 | 33 | 235,000 |

[a]Life expectancy and costs are discounted at 3% per year.
[b]Strategies are ranked by effectiveness, from the least to the most effective, for each age group.
[c]All incremental values compare an alternative with the next most effective strategy (e.g., cost [screen/vaccinate] – cost [no vaccine] = incremental cost [screen/vaccinate over no vaccine]).
[d]Negative values reflect cost savings compared to the next most effective strategy.
[e]Cost-effectiveness ratio: refer to online Appendix I for formula. A strategy dominates if it is both more effective and less expensive than all comparison strategies.
Screen/vaccinate = vaccination of susceptible persons identified through a screening skin test; vaccinate = vaccination of all persons

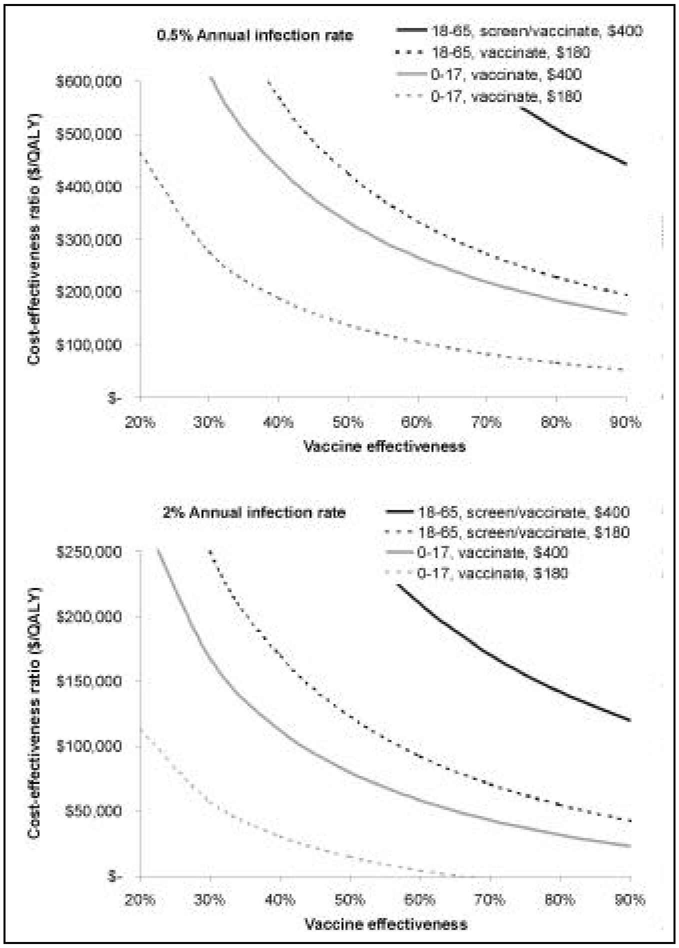

Figure 3. Sensitivity to infection rate, vaccine cost, and vaccine effectiveness. The vaccine is less cost-effective at higher vaccine cost, lower vaccine effectiveness and lower annual infection rates. When the line crosses the x-axis, the strategy is cost-saving. QALY = quality-adjusted life year; screen/vaccinate = vaccination of susceptible persons identified through a screening skin test; vaccinate = vaccination of all persons.

younger populations. A $400 vaccine costs approximately $50,000 per QALY gained among children at 65% effectiveness. A $400 vaccine would be economically unfavorable for the screening/vaccination strategy in adults at an annual infection rate of 2%, even at 90% vaccine effectiveness. At an infection rate of 0.5% per year, as might be seen in southern California (34), all strategies except $180 vaccination at 90% effectiveness in children are economically unfavorable.

### Discount Rate

Among children, vaccination saved 5.95 QALD and $563 over no vaccination when costs and benefits were not discounted. At a discount rate of 5%, vaccination was no longer cost-saving at $27,600 per QALY gained. For adults, changing the discount rate from 0% to 5% changed the cost-effectiveness ratio of screening/vaccination compared with no vaccination from $30,800 to $155,000 per QALY gained.

### Dissemination Rate

Our base-case analysis used a dissemination rate of 0.38% based on the assumption that only blacks and Asians had higher rates of dissemination than whites. We evaluated a range of 0.25% to 0.55% to determine the effect of assuming all non-whites (including Hispanics) had rates of dissemination equal to that of whites (0.25%) or that of blacks (3.4%). Among children, vaccination saved 2.71 QALD and $143 per child vaccinated at an overall dissemination rate of 0.55%, and 1.22 QALD at a cost of $15,300 per QALY gained at the 0.25% dissemination rate. Among adults, varying the dissemination rate from 0.25% to 0.55% changed the cost-effectiveness ratio of screening/vaccination over no vaccination from $25,400 to $125,000 per QALY gained.

### New Residents

If one considers new residents separately, vaccination saved 2.18 QALD and $75 per immigrant child vaccinated, and saved 1.13 QALD per immigrant adult vaccinated, costing $13,500 per QALY gained.

We present best- and worst-case scenarios in Appendix I (online).

### Discussion

We evaluated the usefulness of a potential vaccine against *C. immitis* in highly disease-endemic regions. Vaccination was cost-saving among children. A screening/vaccination strategy cost $62,000 per QALY gained among adults. Although the increase in quality-adjusted life expectancy from an immunization program is modest for one individual patient, this aggregates to an important number of illnesses and deaths prevented (Table 1). Furthermore, the life expectancy gains are comparable with gains from other immunizations. Vaccination for coccidioidomycosis (universally among children and after screening in adults) saved 0.14 to 0.47 life days and 0.53 to 1.86 QALD per person over no vaccination. In comparison, vaccination against pneumococcal bacteremia among elderly people saves 1.2 QALD per person vaccinated (35); infant vaccinations against measles, mumps, rubella, and pertussis each save 2.7, 3, 0.3, and 3.3 life days, respectively (36). To further place benefits in perspective, if the birth cohort in highly endemic counties of California and Arizona were immunized in 2001, 11 deaths would be averted and $3 million would be saved (in net present value) over the lifetime of these children.

The only previous analysis of the cost-effectiveness of a vaccine against coccidioidomycosis drew different conclusions. An IOM report considered the costs and benefits of research and development into a vaccine for *C. immitis* and found that the immunization of infants in endemic regions and immigrants of any age would cost >$100,000 per QALY (14). Our analysis evaluated a different question: If a vaccine were currently available, would it be cost-effective to immunize people in highly endemic regions? Although the IOM report acknowledged that the committee used rough estimates and a simplified model, the primary reason that it reached a different conclusion was that the cost of vaccine development was included in the IOM model. Vaccine development costs will influence the sale price of the vaccine. Project directors of the Valley Fever Vaccine Project estimate that research and development through phase III clinical trials, supported largely by public and private philanthropic sources, will cost $28 million (G. Rutherford, pers. comm.). This figure is 10 times lower than that used by the IOM in its analysis. To recoup $28 million over 5 years by immunizing 60% of the 90,000 annual birth cohort in highly endemic regions of California and Arizona, the vaccine would have to be priced at $100 per vaccinated person. This is less than the $180 vaccine cost assumed in our base-case analysis.

Sensitivity analyses found that the vaccine would be cost-effective in children under assumptions of waning immunity and would be even more effective and cost-saving in children and cost-effective in adults if the vaccine prevented primary infection. In highly endemic regions, vaccination of children is cost-effective even at relatively low levels of vaccine effectiveness if the vaccine is priced at $180. A $180 vaccine had to be >85% effective for screening/vaccination to cost ≤$50,000 per QALY in adults. Paradoxically, an even higher annual infection rate would make a screening/vaccination strategy less favorable in adults because most of them would have already acquired natural immunity by age 40. At an annual infection rate one-fourth of that seen in the Central Valley of California, such as might be seen in southern California, the only cost-effective strategy would be to immunize children with an inexpensive and highly efficacious vaccine. If dissemination rates are higher than our base case, as might be seen in the elderly and those with chronic diseases, screening/vacccination becomes cost-effective for adults.

The vaccine is much more effective in persons without naturally acquired immunity. A vaccination strategy that targeted new residents in highly endemic regions would exploit easily obtainable risk factor information. However, such a policy might be unacceptable to members of the population excluded by such a strategy. Finally, because vaccination is a preventive intervention that can take years before accruing a health benefit (in contrast to acute health-care interventions), our results were highly sensitive to discount rate.

Our model has limitations. The ecology of endemic regions has changed substantially with urbanization since Smith's study in the 1940s (20). Furthermore, his studies documenting infection and dissemination were conducted in the military, which may not be representative of general population exposures. Our base-case analysis used the demographics in highly endemic areas to estimate a composite dissemination rate for a multiracial population. We did not model race independently, nor did we model exposure risk. Workers with high levels of dust exposure may have higher rates of infection and more to gain from early immunization.

Our model assumed that all age groups without prior immunity were at equal risk for adverse health outcomes and had identical health-care costs. However, illness severity and costs may be higher among the elderly. Reported cases of coccidioidomycosis during Arizona's 1991 to 1995 epidemic occurred disproportionately among older adults (11). The study did not discern whether this was due to reporting differences, immune-suppressing coexisting conditions, a preponderance of new immigrants with higher risk for infection, or an independent age-related phenomenon. The death rate from primary pulmonary infection is as high as 26.8% among persons over 65 (37), much higher than our composite base-case estimate of 0.5%. The Kern County data on which we based our cost assumptions revealed that hospitalization, the greatest health-care expenditure associated with acute illness, was 4.2 times more likely among people >50 years old. Thus, we may have underestimated the potential effectiveness and cost savings of the vaccine among older adults.

Finally, our analysis did not model the effects of an immunization program in immunocompromised patients. Persons with compromised cell-mediated immunity, including those with AIDS, malignancy, or therapeutic medical immunosuppression, have higher rates of serious illness. Many of these patients may have reactivation of previous infection; it is unclear whether vaccination would be useful in this clinical scenario. This is an area for future research as results from clinical trials become available.

An uncommon disease in a national context, coccidioidomycosis has substantial ramifications for several regions where epidemics have produced reported annual case rates as high as 15 per 100,000 and substantial economic impact. Diseases affecting <200,000 persons annually (orphan diseases) require more incentives for research and development. Our findings may contribute to the policy decisions for vaccine development and distribution for *C. immitis* (39). Our analysis suggests that a vaccine against *C. immitis* would have substantial public health benefit. An update of this cost-effectiveness analysis can be performed when the results of human vaccine trials become available.

### Acknowledgments

The authors thank George Rutherford, John Galgiani, Royce Johnson, Hans Einstein, John Caldwell, and David Stevens for their expert advice; Janet Morrison and Kristen Jacobs for their library research assistance; and Todd Wagner for his cost- accounting assistance.

Dr. Owens was supported by a Career Development Award from the Department of Veterans Affairs Health Services Research and Development Service. Dr. Barnato was supported by a training grant from the Agency for Healthcare Research and Quality. This work was supported in part by a contract from the California State University Bakersfield Foundation.

Dr. Barnato was a fellow in health care research and policy in the Department of Medicine at Stanford University School of Medicine when she completed this work. She is currently assistant professor of medicine at the University of Pittsburgh School of Medicine. Her research interests include quantitative assessment of preventive interventions and policy tools for quality improvement and cost control in health care.

### References

1. Kirkland TN, Fierer J. Coccidioidomycosis: A reemerging infectious disease. Emerg Infect Dis 1996;2:192-9,
2. Maddy KT. The geographic distribution of *Coccidioides immitis* and possible ecologic implications. Arizona Medicine 1958;15:178.
3. Population estimates. Washington: U.S. Census Bureau; 1999.
4. Flynn NM, Hoeprich PD, Kawachi MM, Lee KK, Lawrence RM, Goldstein E, et al. An unusual outbreak of windborne coccidioidomycosis. N Engl J Med 1979;301:358-61.
5. Schneider E, Hajjeh RA, Spiegel RA, Jibson RW, Harp EL, Marshall GA, et al. A coccidioidomycosis outbreak following the Northridge, Calif, earthquake. JAMA 1997;277:904-8.
6. Johnson WM. Occupational factors in coccidioidomycosis. J Occup Med 1981;23:367-74.
7. Werner SB, Pappagianis D, Heindl I, Mickel A. An epidemic of coccidioidomycosis among archeology students in northern California. N Engl J Med 1972;286:507-12.
8. Standaert SM, Schaffner W, Galgiani JN, Pinner RW, Kaufman L, Durry E, et al. Coccidioidomycosis among visitors to a *Coccidioides immitis*-endemic area: an outbreak in a military reserve unit. J Infect Dis 1995;171:1672-5.

9. Hooper R, Poppell G, Curley R, Husted S, Schillaci R. Coccidioidomycosis among military personnel in Southern California. Mil Med 1980;145:620-3.
10. Centers for Disease Control and Prevention. Coccidioidomycosis–California, 1991-1993. MMWR Morb Mortal Wkly Rep 1994;43:421-3.
11. Centers for Disease Control and Prevention. Coccidioidomycosis–Arizona, 1990-1995. MMWR Morb Mortal Wkly Rep 1996;45:1069-73.
12. Caldwell J, Welch G, Johnson RH, Einstein HE. The economic impact of coccidioidomycosis in Kern County, California, 1991 to 1993. In: Einstein HE, Catanzaro A, editors. Coccidioidomycosis: proceedings of the 5th international conference. Washington: National Foundation for Infectious Diseases; 1994: p. 88-97.
13. Pappagianis D. Evaluation of the protective efficacy of the killed *Coccidioides immitis* spherule vaccine in humans. Am Rev Respir Dis 1993;148:656-60.
14. Stratton KR, Durch JS, Lawrence RS. Vaccines for the 21st century: a tool for decision making. Washington: Division of Health Promotion and Disease Prevention, Institute of Medicine; 1999.
15. Gold M, Franks P, McCoy K, Fryback DG. Toward consistency in cost-utility analyses: using national measures to create condition-specific values. Med Care 1998;36:778-92.
16. Beck JR, Sauker SG. The Markov process in medical prognosis. Med Decis Making 1983;3:419-58.
17. Sonnenberg FA, Beck JR. Markov models in medical decision making: a practical guide. Med Decis Making 1993;13;322-38.
18. Centers for Disease Control and Prevention, National Center for Health Statistics (NCHS). Monthly vital statistics report: annual summary of births, marriages, divorces, and deaths: United States. Hyattsville (MD): NCHS; 1995.
19. Centers for Disease Control and Prevention. Coccidioidomycosis–United States, 1991-1992. MMWR Morb Mortal Wkly Rep 1993; 42:21-4.
20. Smith CE, Beard RR. Varieties of coccidioidal infection in relation to the epidemiology and control of diseases. Am J Public Health 1946;36:1394-402.
21. Einstein HE, Johnson RH. Coccidioidomycosis: new aspects of epidemiology and therapy. Clin Infect Dis 1993;16:349-54.
22. Arsura E, Caldwell J, Johnson R, Einstein H, Welch G, Talbot R, et al. Coccidioidomycosis epidemic of 1991: epidemiologic features. In: Einstein HE, Catanzaro A, editors. 5th International Conference on Coccidioidomycosis. Stanford University, Aug 24-27, 1994. Bethesda (MD): National Foundation for Infectious Diseases; 1996. p. 98-107.
23. Galgiani JN. Coccidioidomycosis: a regional disease of national importance; rethinking approaches for control. Ann Intern Med 1999;130:293-300.
24. Assad S. Over a decade of experience with a yeast recombinant hepatitis B vaccine. Vaccine 1999;18:57-67.
25. Lebowitz MD, Johnson WM, Kaltenborn W. Coccidioidin skin test reactivity and cross-reactivity with histoplasmin in a Tucson population. In: Ajello L, editor. Coccidioidomycosis: current clinical and diagnostic status. Vol 1. Miami: Symposia Specialists; 1977. p. 45-61.
26. Dodge RR, Lebowitz MD, Barbee R, Burrows B. Estimates of *C. immitis* infection by skin test reactivity in an endemic community. Am J Public Health 1985;75:863-5.
27. Geller RD, Maynard JE, Jones V. Coccidioidin sensitivity among Southwestern American Indians. Am Rev Respir Dis 1973;107:301-2.
28. Stevens DA, Levine HB, Deresinski SC, Ten Eyck DR, Restrepo MA. Epidemiological and clinical skin testing studies with spherulin. In: Ajello L, editor. Coccidioidomycosis: current clinical and diagnostic status. Vol 1. Miami: Symposia Specialists; 1977. p. 107-14.
29. Levine HB, Gonzalez-Ochoa A, Ten Eyck DR. Dermal sensitivity to *Coccidioides immitis*. A comparison of responses elicited in man by spherulin and coccidioidin. Am Rev Respir Dis 1973;107:379-86.
30. Levine HB, Restrepon A, Eyck DR, Stevens DA. Spherulin and coccidioidin: cross-reactions in dermal sensitivity to histoplasmin and paracoccidioidin. Am J Epidemiol 1975;101:512-6.
31. Healthcare Cost and Utilization Project: Nationwide Inpatient Sample. Rockville (MD): Agency for Healthcare Research and Quality; 1996.
32. Revised 2000 National physician fee schedule. Baltimore: Health Care Financing Administration; 2000.
33. Manton KG, Etallard E. Chapter 6: Program payment and utilization trends for Medicare beneficiaries with disabilities. In: Wiener JM, Clauser SB, Kenner DL, editors. Persons with disabilities. Washington: The Brookings Institution; 1995. p. 117-62.
34. Klotz AL, Biddle M. Coccidioidin skin test survey of San Fernando Valley State college students over a five year period. In: Ajello L, editor. The Second Symposium on Coccidioidomycosis. Phoenix (AZ): University of Arizona Press; 1965. p. 251-3.
35. Sisk JE, Moskowitz AJ, Whang W, Lin JD, Fedson DS, McBean AM, et al. Cost-effectiveness of vaccination against pneumococcal bacteremia among elderly people. JAMA 1997;278:1333-9.
36. Wright JC, Weinstein MC. Gains in life expectancy from medical interventions–standardizing data on outcomes. N Engl J Med 1998;339:380-6.
37. Arsura EL. The association of age and mortality in coccidioidomycosis [letter]. J Am Geriatr Soc 1997;45:532-3.
38. Lang J, Wood SW. Development of orphan vaccines: an industry perspective. Emerg Infect Dis 1999;5:749-56.

### Appendix I. Clinical and Economic Background

Appendix I is online only; it contains the formula used to calculate the incremental cost-effectiveness of these strategies; URL: http://www.cdc.gov/ncid/eid/vol7/no5/barnato_appendix1.htm

### Appendix II: Model Input Variables

Appendix II Table. Input variables, quality of data, and sources[a]

| Input variable | Base-case estimate (range) | Quality of evidence[b] | Source |
|---|---|---|---|
| Epidemiology (%) | | | |
| Vaccine effectiveness | 75 (20-90) | I | 2 |
| Skin-test sensitivity | 70 (50-80) | II-2 | 3-5 |
| Skin-test specificity | 90 (70-97) | II-2 | 4,6,7 |
| Annual infection rate | 2 (0.25-3) | II-3 | 5,8-16 |
| Annual emigration among vaccinees out of highly endemic region | 0.5 (0-4.2) | II-2, III | [c] |
| Symptomatic primary pulmonary disease after infection | 40 | II-2 | 18 |
| Diagnosed symptomatic primary pulmonary disease | 10 (5-15) | III | d |
| Death from primary pulmonary disease, given diagnosis | 0.5 (0-26) | II-2 | 19-22 |
| Chronic pulmonary disease after diagnosed primary infection | 5 (1-10) | III | 23-26 |
| Death from chronic pulmonary disease | 5 (0-20) | III | 24[e] |
| Dissemination after infection | 0.38(0.25-0.55) | II-2 | 17 |
| Meningitis, given dissemination | 33 (23-44) | II-2 | 21,26 |
| Death from meningeal dissemination | 7 (5-40) | II-2, III | 27[d,e] |
| Moderate disability after meningeal dissemination | 50 (40-60) | III | 27[d,e] |
| Severe disability after meningeal dissemination | 17 (10-30) | III | 27[d,e] |
| Annual meningeal dissemination relapse rate, on treatment | 2 (0-5) | I, II-2 | 28-30 |
| Death from nonmeningeal dissemination | 2 (0-10) | III | [e] |
| Moderate disability after nonmeningeal dissemination | 33 (20-50) | III | [d,e] |
| Annual nonmeningeal dissemination relapse rate | | | |
| On treatment | 2 (0-5) | I, II-2, III | 4[c] |
| Off treatment | 50 (35-65) | I, II-2, III | 31-34[c] |
| Mild vaccine side effects | 25 (10-40) | II-2 | 35 |
| Vaccine anaphylaxis, x $10^{-4}$ | 1.67 (0.1-10) | II-2 | 35 |
| Direct medical costs ($) | | | |
| Three doses of vaccine | 180 (100-400) | III | 36,37 |
| Skin test | 12 (9-15) | III | 38 |
| Home care, per month | 2,450 (1,840-3,060) | II-2 | 39 |
| Diagnosed pulmonary disease | 2,090 (1,570-2,610) | II-2, III | 40 |
| Incident meningeal dissemination | 9,510 (7,130-11,890) | II-2 | 40 |
| Medication and follow-up after *Coccidioides immitis* meningitis,[f] per month | 1,510 (1,130-1,890) | II-2 | 41[e,g] |
| Incident nonmeningeal dissemination | 6,950 (5,210-8,690) | II-2 | 40 |
| Medication and follow-up for chronic pulmonary infection and nonmeningeal dissemination,[f] per month | 530 (290-790) | II-2 | 41[e,g] |
| Inpatient vaccine anaphylaxis treatment | 2,180 (1,640-2,730) | II-2 | 40 |

Appendix II Table. Input variables, quality of data, and sources[a] (continued)

| Input variable | Base-case estimate (range) | Quality of evidence[b] | Source |
|---|---|---|---|
| Time costs[h] | | | |
| Average wage ($ per hour) | 12 (9-15) | II-2 | [d] |
| Average clinic visit (hours) | 1.25 (0.5-2) | III | Assumed |
| Lost work due to undiagnosed primary pulmonary disease (days) | 5 (0-10) | III | Assumed |
| For parents of sick children (days) | 3 (0-5) | III | Assumed |
| Utilities | | | |
| Well | 0.94 to 0.70[i] | II-2 | 42 |
| Diagnosed primary pulmonary infection | 0.90 (0.85-0.95) | III | [d] |
| Chronic pulmonary infection (proxy, pulmonary tuberculosis) | 0.57 (0.29-0.84) | II-2, III | 42 |
| Meningeal dissemination (proxy, paraplegia) | 0.40 (0.21-0.52) | II-2, III | 42 |
| Nonmeningeal dissemination (proxy, orthopedic impairment) | 0.59 (0.34-0.84) | II-2, III | 42 |
| Severe disability after meningitis (proxy, hemiplegia) | 0.27 (0.10-0.38) | II-2, III | 42 |
| Moderate disability after meningitis (proxy, sciatica) | 0.72 (0.52-0.92) | II-2, III | 42 |
| Moderate disability after nonmeningeal dissemination (proxy, arthritis) | 0.69 (0.51-0.92) | II-2, III | 42 |
| Chronic azole treatment (proxy, warfarin treatment) | 0.98 (0.92-1.0) | II-2, III | 43 |
| Dead | 0 | III | Assumed |
| Vaccine side effect quality-of-life decrement (days) | 0.1 (0-0.2) | III | Assumed |
| Other variables (%) | | | |
| Discount rate | 3 (0-5) | III | 44 |

[a]The base-case estimate represents our best estimate for each value. All costs are in 2000 U.S. dollars.
[b]The quality rating is derived from the U.S. Preventive Services Task Force Guide to Clinical Preventive Services (1). Source of evidence: I: at least one properly randomized controlled trial; II-1: well-designed controlled trial without randomization; II-2: well-designed cohort or case-control analytic studies; II-3: multiple time series with or without intervention; III: opinions of respected authorities; descriptive studies and case reports; or reports of expert committees (1).
[c]Internal Revenue Service, unpub. data.
[d]John Galgiani, pers. comm.
[e]Hans Einstein, pers. comm.
[f]We assumed that meningitis patients were treated with 800 mg of daily fluconazole, and chronic pulmonary and nonmeningeal dissemination patients with either 400 mg fluconazole or 400 mg ketoconazole daily (Royce Johnson, pers. comm., 1999). A 50:50 distribution of fluconazole and ketoconazole use represents our base case; the upper end of the range assumes all nonmeningeal dissemination patients receive fluconazole in follow-up, whereas the lower end assumes they receive the less expensive ketoconazole.
[g]Ron Talbot, pers. comm.
[h]Based on a weighted adjusted gross income of $24,105 for taxpayers in the 10 highly endemic counties (Internal Revenue Service, unpub. data).
[i]Mean HALex scores for healthy persons, by age group (when men and women had differing mean scores, we chose the higher of the two scores): <5=0.94; 5-17=0.93; 18-24= 0.92; 25-34=0.91; 35-44=0.90; 45-54=0.87; 55-64=0.81; 65-74=0.78; >75=0.70 (43).

### References

1. U.S. Preventive Services Task Force. Guide to clinical preventive services. 2nd ed. Alexandria (VA): International Medical Publishing; 1996.
2. Szmuness W, Stevens CE, Harley EJ, Zang EA, Oleszko WR, William DC, et al. Hepatitis B vaccine: demonstration of efficacy in a controlled clinical trial in a high risk population in the United States. N Engl J Med 1980;303:833-41.
3. Stevens DA, Levine HB, Deresinski SC, Ten Eyck DR, Restrepo MA. Epidemiological and clinical skin testing studies with spherulin. In: Ajello L, editor. Coccidioidomycosis: Current clinical and diagnostic status. Vol. 1. Miami: Symposia Specialists; 1977. p. 107-14.
4. Lebowitz MD, Johnson WM, Kaltenborn W. Coccidioidin skin test reactivity and cross-reactivity with histoplasmin in a Tucson population. In: Ajello L, editor. Coccidioidomycosis: Current clinical and diagnostic status. Vol. 1. Miami: Symposia Specialists; 1977. p. 45-61.
5. Dodge RR, Lebowitz MD, Barbee R, Burrows B. Estimates of *C. immitis* infection by skin test reactivity in an endemic community. Am J Public Health 1985;75:863-5.
6. Levine HB, Gonzalez-Ochoa A, Ten Eyck DR. Dermal sensitivity to *Coccidioides immitis*. A comparison of responses elicited in man by spherulin and coccidioidin. Am Rev Respir Dis 1973;107:379-86.
7. Levine HB, Restrepon A, Eyck DR, Stevens DA. Spherulin and coccidioidin: cross-reactions in dermal sensitivity to histoplasmin and paracoccidioidin. Am J Epidemiol 1975;101:512-6.
8. Gifford M, Buss W, Douds R. Annual report of the Kern County Health Department for the fiscal year July 1, 1936 to June 30, 1937.
9. Edwards PQ, Palmer CE. Prevalence of sensitivity to coccidioidin, with special reference to specific and nonspecific reactions to coccidioidin and to histoplasmin. Diseases of the Chest 1957;31:35-60.
10. Hugenhotz PG. Skin test survey at Williams Air Force Base, Arizona. In: Symposium on Coccidioidomycosis, Phoenix, AZ. Washington: U.S. Government Printing Office; 1957.
11. Maddy KT. The geographic distribution of *Coccidioides immitis* and possible ecologic implications. Arizona Medicine 1958;15:178.
12. Klotz AL, Biddle M. Coccidioidin skin test survey of San Fernando Valley State college students over a five year period. In: The Second Symposium on Coccidioidomycosis. Phoenix (AZ): University of Arizona Press; 1965.
13. Doto I. Coccidioidin, histoplasmin, and tuberculin sensitivity among school children in Maricopa Co., Arizona. Am J Epidemiol 1972;95:464.
14. Catanzaro A. Coccidioidin sensitivity in San Diego schools. Sabouraudia 1979;17:85-9.

15. Fredrich BE. A skin test survey of valley fever in Tijuana, Mexico. Soc Sci Med 1989;29:1217-27.
16. Larwood T. Coccidioidin skin testing in Kern County, California: decrease in infection rate over 58 years. Clin Infect Dis 2000;30:612-3.
17. Smith CE, Beard RR. Varieties of coccidiodal infection in relation to the epidemiology and control of diseases. Am J Public Health 1946;36:1394-402.
18. Bayer AS. Fungal pneumonias; pulmonary coccidioidal syndromes (Part 1). Primary and progressive primary coccidioidal pneumonias --diagnostic, therapeutic, and prognostic considerations. Chest 1981;79:575-83.
19. Arsura E, Caldwell J, Johnson R, Einstein H, Welch G, Talbot R, et al. Coccidioidomycosis epidemic of 1991: epidemiologic features. In: 5th International Conference on Coccidioidomycosis. Stanford (CA) University: National Foundation for Infectious Diseases; 1994.
20. Arsura EL. The association of age and mortality in coccidioidomycosis Letter. J Am Geriatr Soc 1997;45:532-3.
21. Johnson RH, Caldwell JW, Welch G, Einstein HE. The great coccidioidomycosis epidemic: clinical features. In: Coccidioidomycosis: 5th International Conference. Stanford (CA) University: National Foundation for Infectious Diseases; 1994.
22. Sarosi GA, Parker JD, Doto IL, Tosh FE. Chronic pulmonary coccidioidomycosis. N Engl J Med 1970;283:325-9.
23. Bayer AS. Fungal pneumonias: pulmonary coccidioidal syndromes (Part 2). Miliary, nodular, and cavitary pulmonary coccidioidomycosis; chemotherapeutic and surgical considerations. Chest 1981;79:686-91.
24. Einstein HE, Johnson RH. Coccidioidomycosis: new aspects of epidemiology and therapy. Clin Infect Dis 1993;16:349-54.
25. Galgiani JN. Coccidioidomycosis. In: Remington JS, Swartz MN, editors. Curr Clin Top Infect Dis 1997:188-204.
26. Vincent T, Galgiani John N, Huppert M, Salkin D. The natural history of coccidioidal meningitis: VA-Armed Forces Cooperative Studies, 1955-1958. Clin Infect Dis 1993;16:247-54.
27. Bouza E, Dreyer JS, Hewitt WL, Meyer RD. Coccidioidal meningitis. An analysis of thirty-one cases and review of the literature. Medicine 1981;60:139-72.
28. Galgiani JN, Catanzaro A, Cloud GA, Higgs J, Friedman BA, Larsen RA, et al. Fluconazole therapy for coccidioidal meningitis. The NIAID-Mycoses Study Group. Ann Intern Med 1993;119:28-35.
29. Perez JA Jr, Johnson RH, Caldwell JW, Arsura EL, Nemecheck P. Fluconazole therapy in coccidioidal meningitis maintained with intrathecal amphotericin B. Arch Intern Med 1995;155:1665-8.
30. Tucker RM, Denning DW, Dupont B, Stevens DA. Itraconazole therapy for chronic coccidioidal meningitis. Ann Intern Med 1990;112:108-12.
31. Catanzaro A, Galgiani JN, Levine BE, Sharkey-Mathis PK, Fierer J, Stevens DA, et al. Fluconazole in the treatment of chronic pulmonary and nonmeningeal disseminated coccidioidomycosis. NIAID Mycoses Study Group. Am J Med 1995;98:249-56.
32. Galgiani JN, Catanzaro A, Cloud GA, Johnson RH, Williams PL, Mirels LF, et al. Comparison of oral fluconazole and itraconazole for progressive, nonmeningeal coccidioidomycosis. A randomized, double-blind trial. Mycoses Study Group. Ann Intern Med 2000;133:676-86.
33. Graybill JR, Stevens DA, Galgiani JN, Dismukes WE, Cloud GA. Itraconazole treatment of coccidioidomycosis. NAIAD Mycoses Study Group. Am J Med 1990;89:282-90.
34. Oldfield EC 3rd, Bone WD, Martin CR, Gray GC, Olson P, Schillaci RF. Prediction of relapse after treatment of coccidioidomycosis. Clin Infect Dis 1997;25:1205-10.
35. Niu MT, Rhodes P, Salive M, Liveley T, Davis DM, Black S, et al. Comparative safety data of two recombinant hepatitis B vaccines in children: data from the Vaccine Adverse Event Reporting System (VAERS) and Vaccine Safety Datalink (VSD). J Clin Epidemiol 1998;51:503-10.
36. Andre FE. Summary of safety and efficacy data on a yeast derived hepatitis B vaccine. Am J Med 1989;87:14s-20s.
37. Assad S. Over a decade of experience with a yeast recombinant hepatitis B vaccine. Vaccine 1999;18:57-67.
38. Revised 2000 National Physician Fee Schedule. Baltimore: Health Care Financing Administration; 2000.
39. Manton KG, Etallard E. Chapter 6: Program payment and utilization trends for Medicare beneficiaries with disabilities. In: Wiener JM, Clauser SB, Kenner DL, editors. Persons with disabilities. Washington: The Brookings Institution; 1995. p. 117-62.
40. Healthcare Cost and Utilization Project. Nationwide inpatient sample. Rockville (MD): Agency for Healthcare Research and Quality; 1996.
41. Wholesale acquisition prices for pharmaceuticals in the United States. Montvale (NJ): Medical Economics; 1999.
42. Gold M, Franks P, McCoy K, Fryback DG. Toward consistency in cost-utility analyses: using national measures to create condition-specific values. Med Care 1998;36:778-92.
43. Gage B, Cardinalli A, Owens D. The effect of stroke and stroke prophylaxis with aspirin or warfarin on quality of life. Arch Intern Med 1996;156:1829-36.
44. Lipscomb J, Weinstein MC, Torrance GW. Time Preference. In: Gold MR, Siegel JE, Russell LB, Weinstein MC, editors. Cost-effectiveness in health and medicine. New York: Oxford University Press; 1996. p. 214-35.
